# Supplementary material for: A yeast phenomic model for the influence of Warburg metabolism on genetic buffering of doxorubicin
Source: Cancer Metab. 2019 Oct 23;7:9. doi: 10.1186/s40170-019-0201-3 (PMC6806529; doi:10.1186/s40170-019-0201-3)
Supplement: Supplementary file 9 — Additional file 9. GO term-specific heatmaps for REMc/GTF-enriched clusters. GO term-specific heatmaps for significant GO process terms were generated as described in methods and Figs. 3 and 4. Any related child terms are presented in subsequent pages of the parent file name. GO terms with more than 100 children, with 2 or fewer genes annotated to the term, or a file size over 300KB are not shown. All heatmaps are generated with the same layout (see Figs. 3 and 4). [file 40170_2019_201_MOESM9_ESM.bz2 › Additional_File9_GOTermHeatmaps/Additional_File9_GOTermHeatmap/protein-DNA_complex_assembly.pdf]

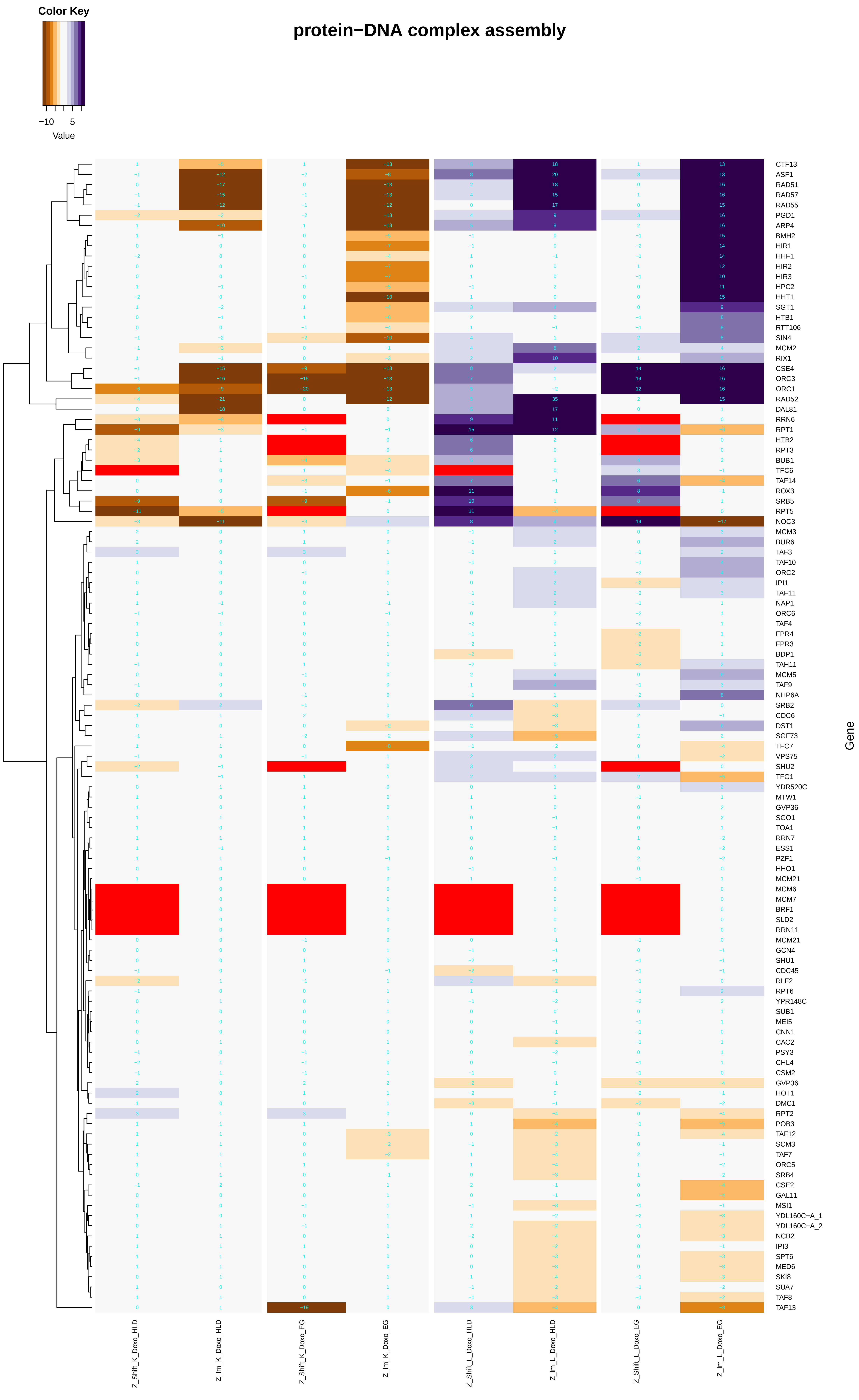
protein−DNA complex assembly
Color Key
−10
5
Value
CTF13
ASF1
RAD51
RAD57
RAD55
PGD1
ARP4
BMH2
HIR1
HHF1
HIR2
HIR3
HPC2
HHT1
SGT1
HTB1
RTT106
SIN4
MCM2
RIX1
CSE4
ORC3
ORC1
RAD52
DAL81
RRN6
RPT1
HTB2
RPT3
BUB1
TFC6
TAF14
ROX3
SRB5
RPT5
NOC3
MCM3
BUR6
TAF3
TAF10
ORC2
IPI1
TAF11
NAP1
ORC6
TAF4
FPR4
FPR3
BDP1
TAH11
MCM5
TAF9
NHP6A
SRB2
CDC6
DST1
SGF73
TFC7
VPS75
SHU2
TFG1
YDR520C
MTW1
GVP36
SGO1
TOA1
RRN7
ESS1
PZF1
HHO1
MCM21
MCM6
MCM7
BRF1
SLD2
RRN11
MCM21
GCN4
SHU1
CDC45
RLF2
RPT6
YPR148C
SUB1
MEI5
CNN1
CAC2
PSY3
CHL4
CSM2
GVP36
HOT1
DMC1
RPT2
POB3
TAF12
SCM3
TAF7
ORC5
SRB4
CSE2
GAL11
MSI1
YDL160C−A_1
YDL160C−A_2
NCB2
IPI3
SPT6
MED6
SKI8
SUA7
TAF8
TAF13
Gene
Z_Shift_K_Doxo_HLD
Z_Im_K_Doxo_HLD
Z_Shift_K_Doxo_EG
Z_Im_K_Doxo_EG
Z_Shift_L_Doxo_HLD
Z_Im_L_Doxo_HLD
Z_Shift_L_Doxo_EG
Z_Im_L_Doxo_EG

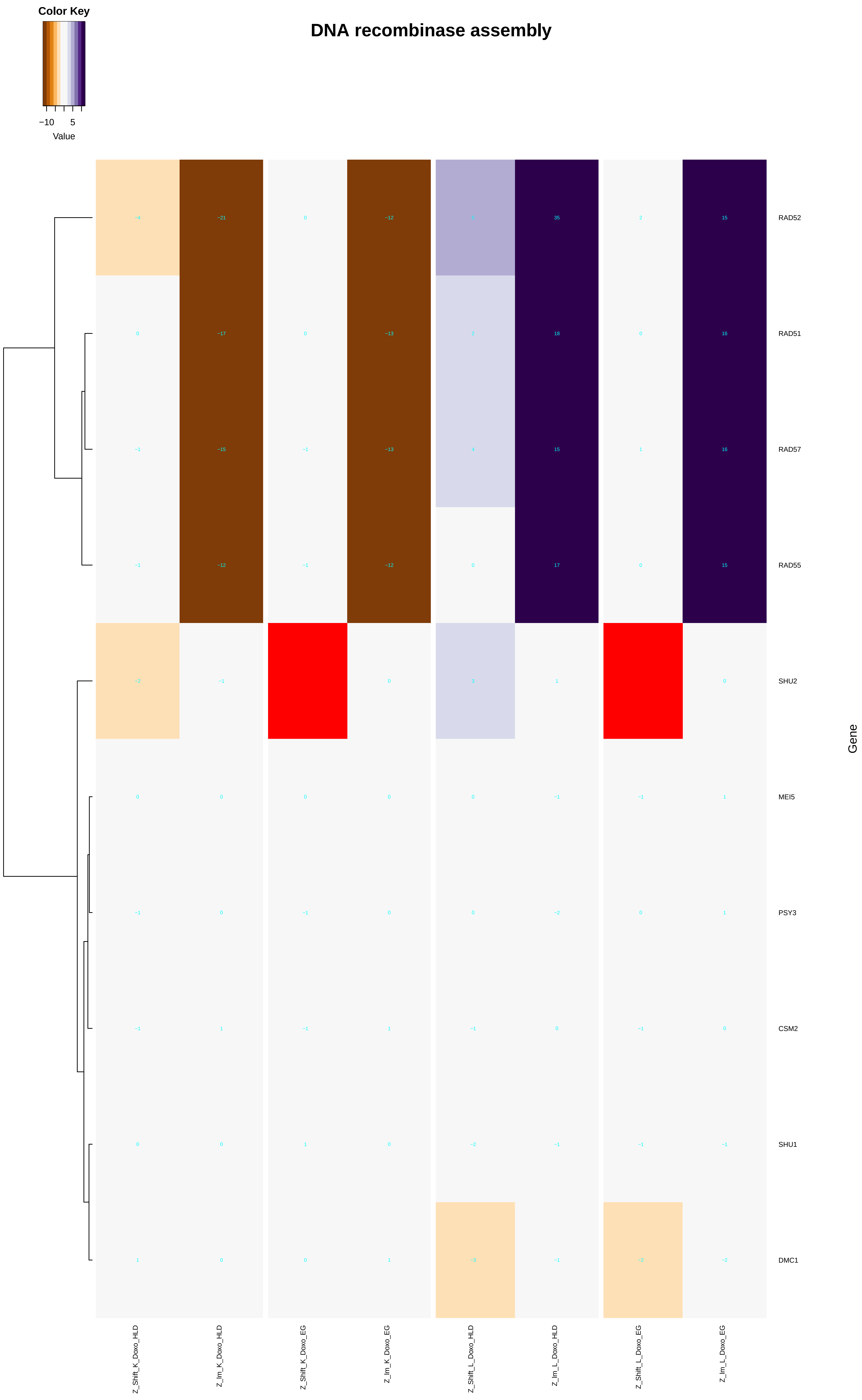

Color Key
−10
5
Value
DNA recombinase assembly
RAD52
RAD51
RAD57
RAD55
SHU2
MEI5
PSY3
CSM2
SHU1
DMC1
Gene
Z_Shift_K_Doxo_HLD
Z_lm_K_Doxo_HLD
Z_Shift_K_Doxo_EG
Z_lm_K_Doxo_EG
Z_Shift_L_Doxo_HLD
Z_lm_L_Doxo_HLD
Z_Shift_L_Doxo_EG
Z_lm_L_Doxo_EG

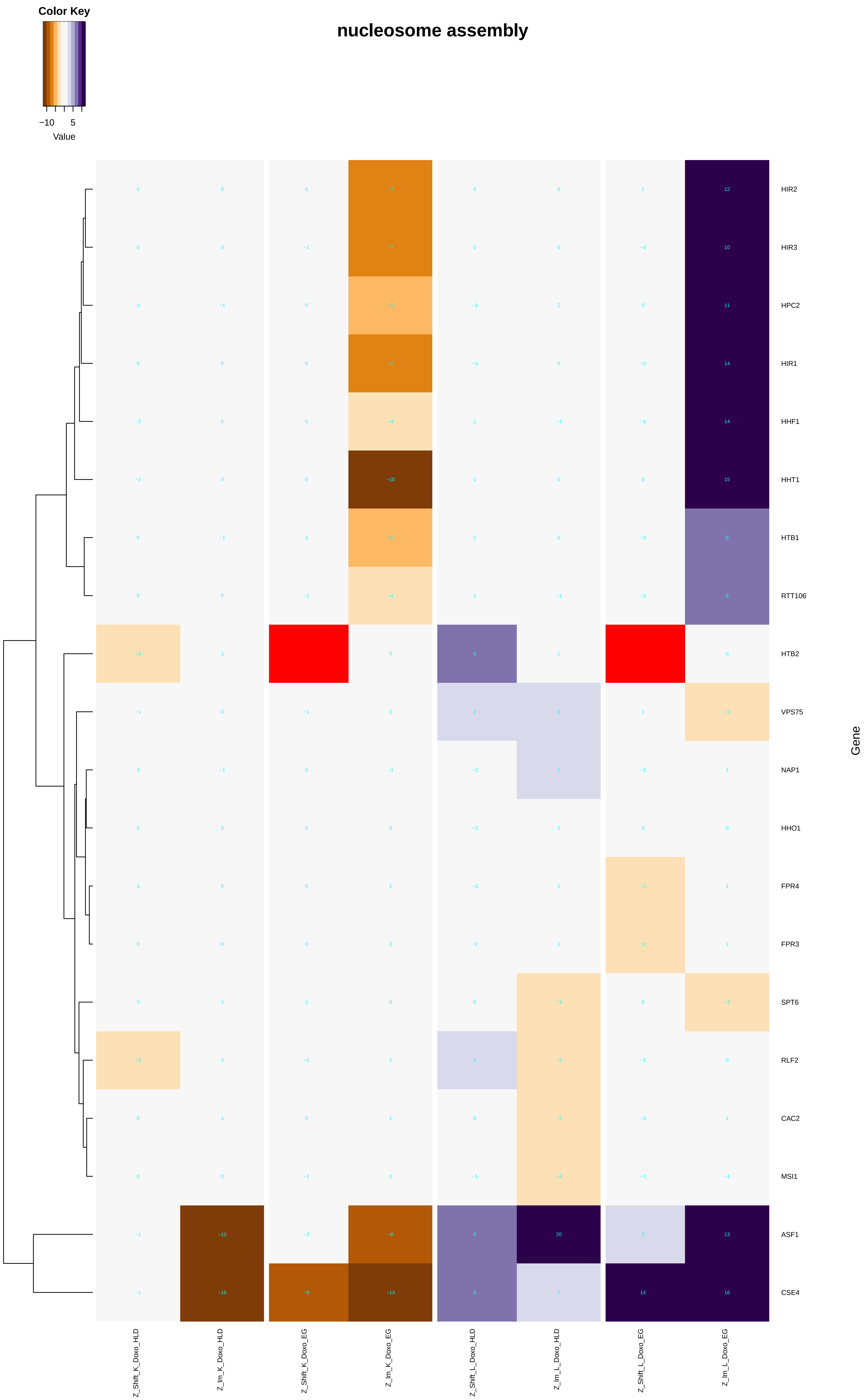
Color Key
−10
5
Value
nucleosome assembly
HIR2
HIR3
HPC2
HIR1
HHF1
HHT1
HTB1
RTT106
HTB2
VPS75
NAP1
HHO1
FPR4
FPR3
SPT6
RLF2
CAC2
MSI1
ASF1
CSE4
Gene
Z_Shift_K_Doxo_HLD
Z_Im_K_Doxo_HLD
Z_Shift_K_Doxo_EG
Z_Im_K_Doxo_EG
Z_Shift_L_Doxo_HLD
Z_Im_L_Doxo_HLD
Z_Shift_L_Doxo_EG
Z_Im_L_Doxo_EG

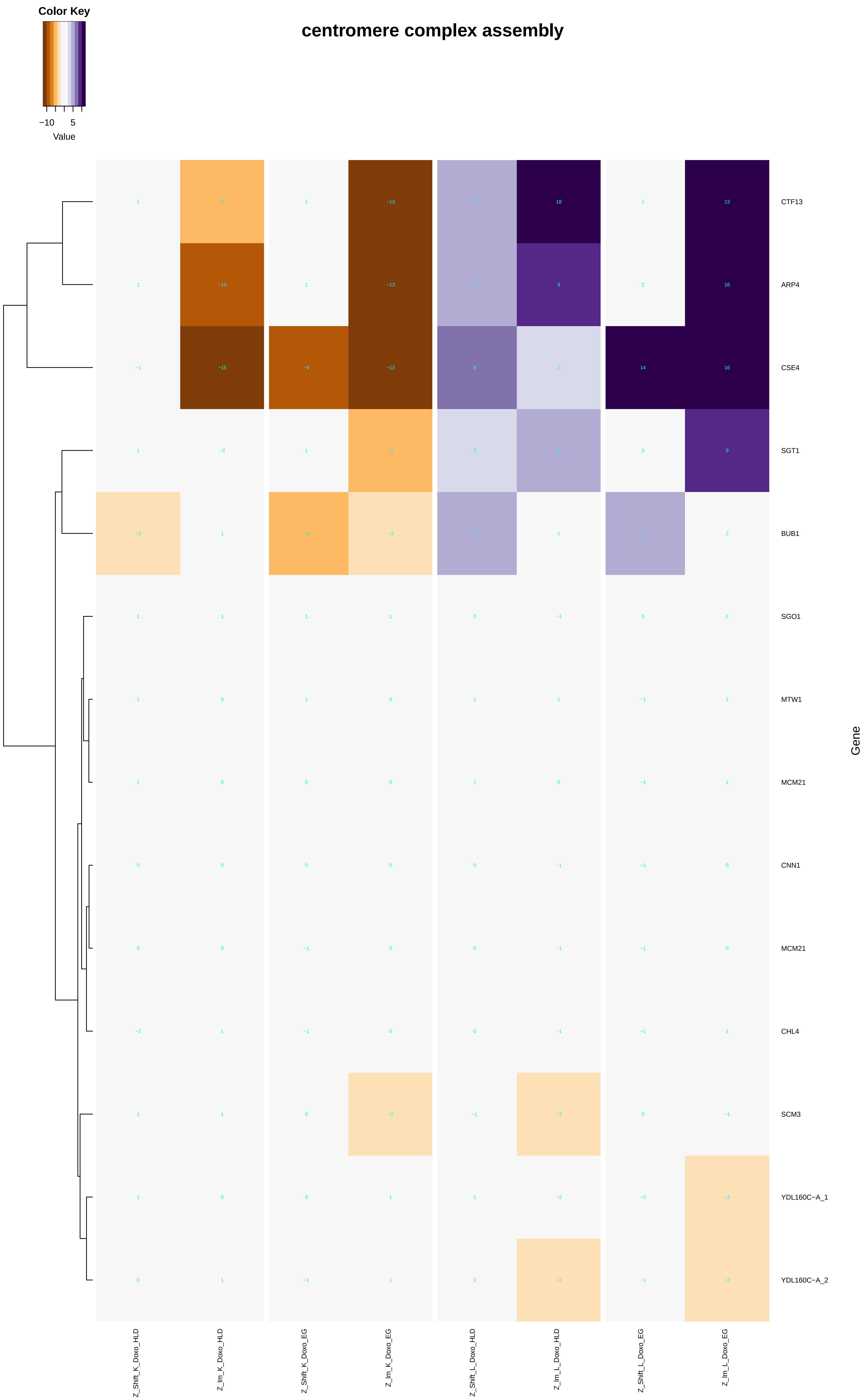
Color Key
−10
5
Value
centromere complex assembly
CTF13
ARP4
CSE4
SGT1
BUB1
SGO1
MTW1
MCM21
CNN1
MCM21
CHL4
SCM3
YDL160C−A_1
YDL160C−A_2
Gene
Z_Shift_K_Doxo_HLD
Z_Im_K_Doxo_HLD
Z_Shift_K_Doxo_EG
Z_Im_K_Doxo_EG
Z_Shift_L_Doxo_HLD
Z_Im_L_Doxo_HLD
Z_Shift_L_Doxo_EG
Z_Im_L_Doxo_EG

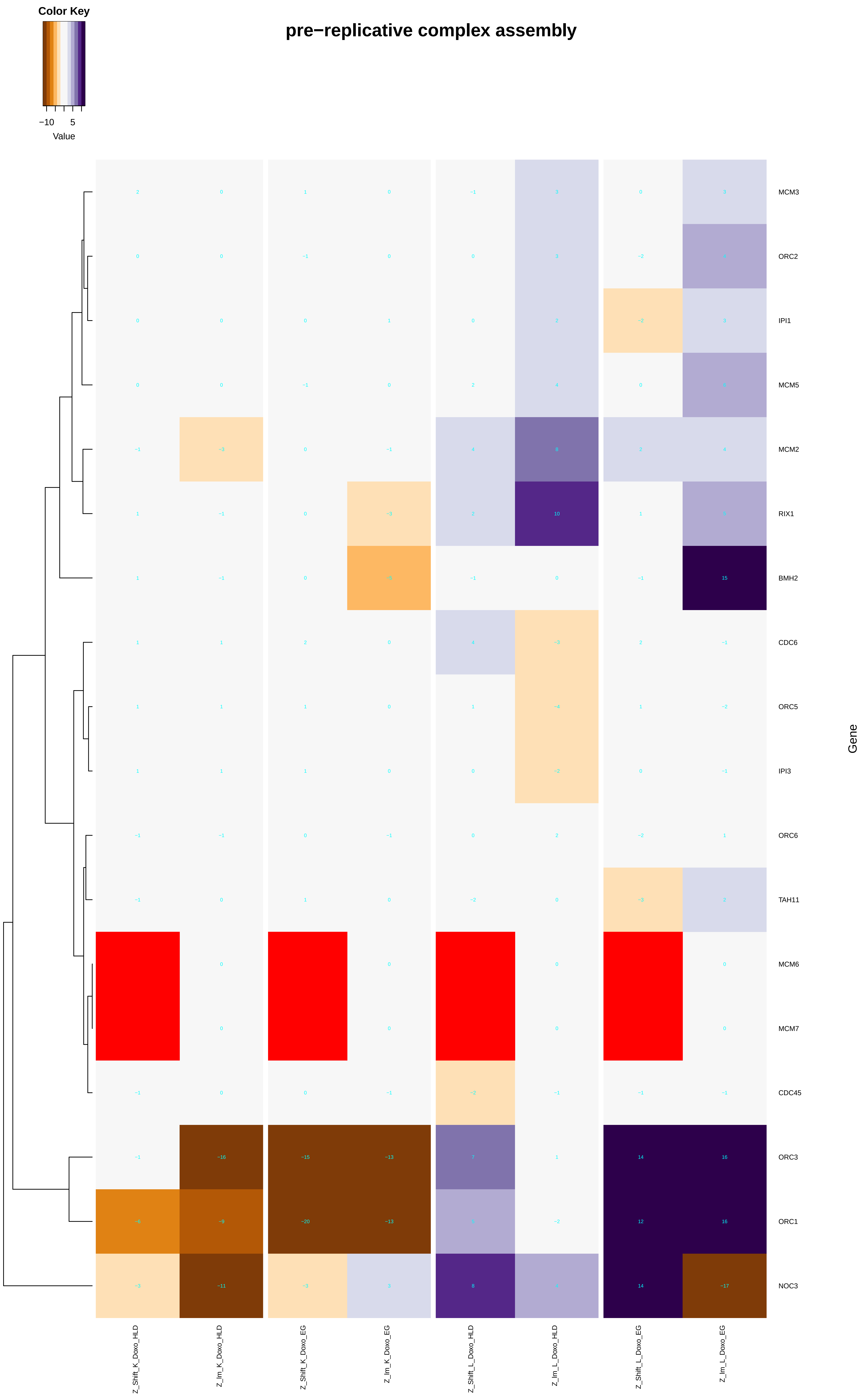

Color Key
−10
5
Value
pre−replicative complex assembly
MCM3
ORC2
IPI1
MCM5
MCM2
RIX1
BMH2
CDC6
ORC5
IPI3
ORC6
TAH11
MCM6
MCM7
CDC45
ORC3
ORC1
NOC3
Gene
Z_Shift_K_Doxo_HLD
Z_Im_K_Doxo_HLD
Z_Shift_K_Doxo_EG
Z_Im_K_Doxo_EG
Z_Shift_L_Doxo_HLD
Z_Im_L_Doxo_HLD
Z_Shift_L_Doxo_EG
Z_Im_L_Doxo_EG

DNA−templated transcriptional preinitiation complex assembly

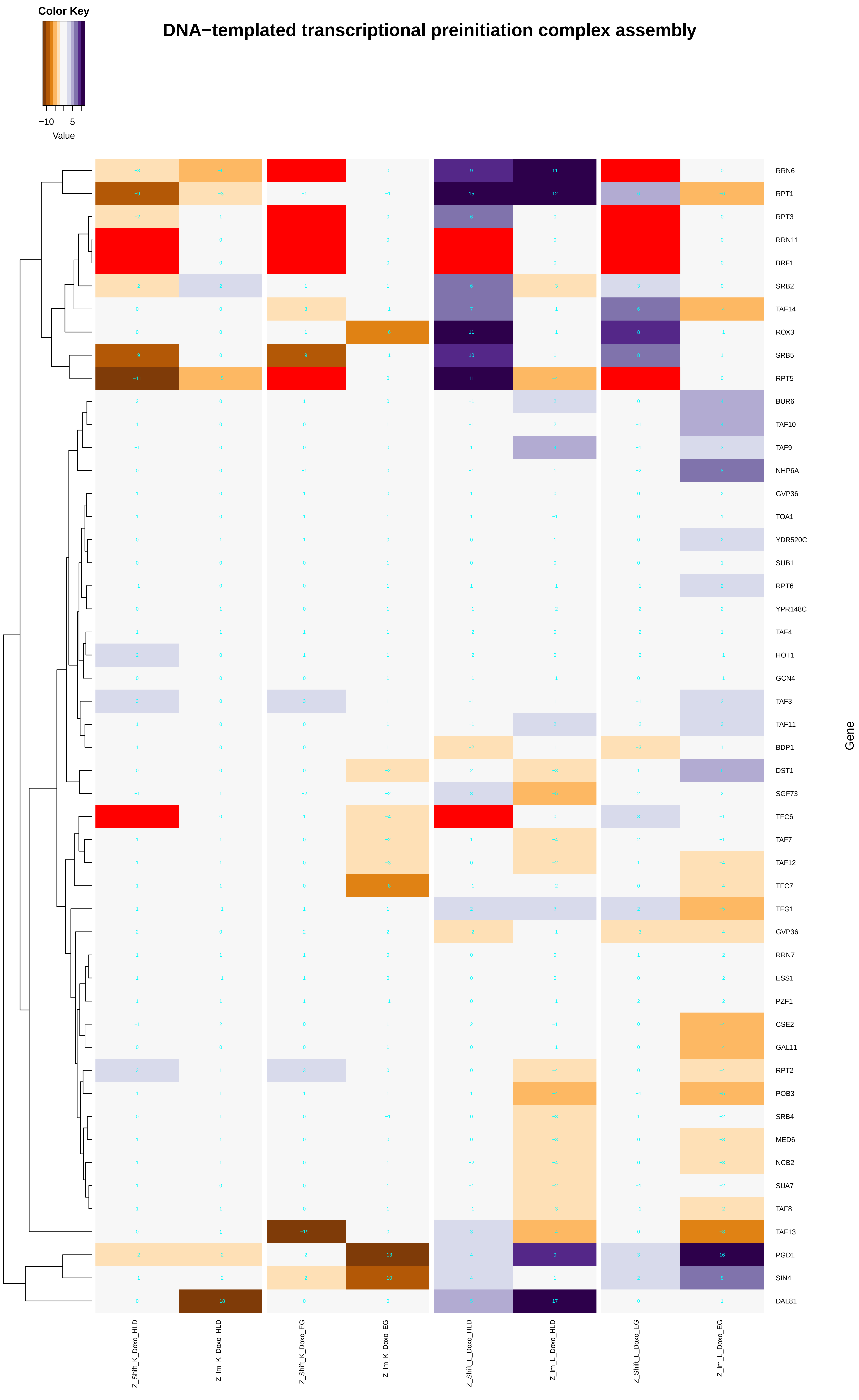

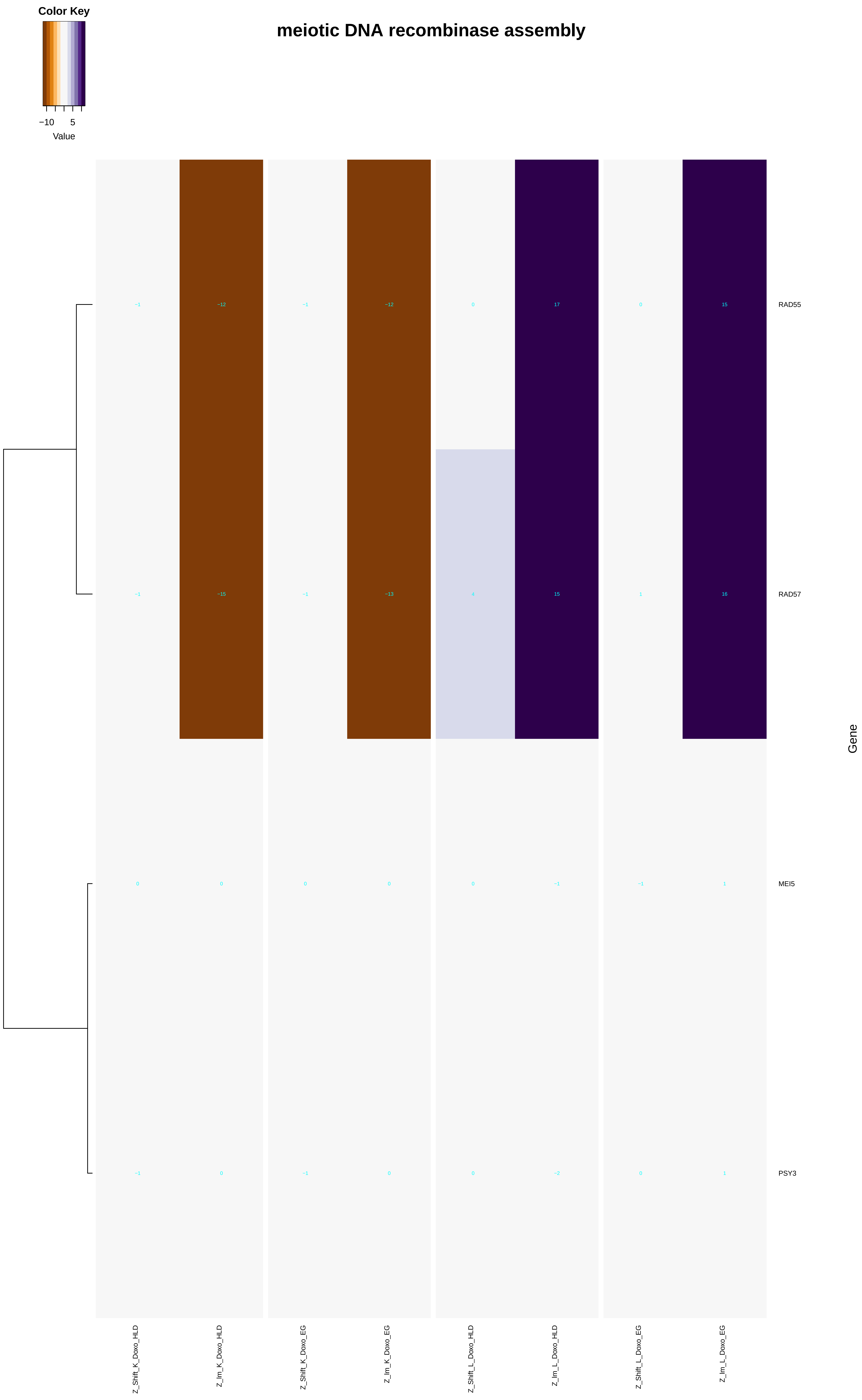

meiotic DNA recombinase assembly
Color Key
−10
5
Value
RAD55
RAD57
MEI5
PSY3
Gene
Z_Shift_K_Doxo_HLD
Z_Im_K_Doxo_HLD
Z_Shift_K_Doxo_EG
Z_Im_K_Doxo_EG
Z_Shift_L_Doxo_HLD
Z_Im_L_Doxo_HLD
Z_Shift_L_Doxo_EG
Z_Im_L_Doxo_EG

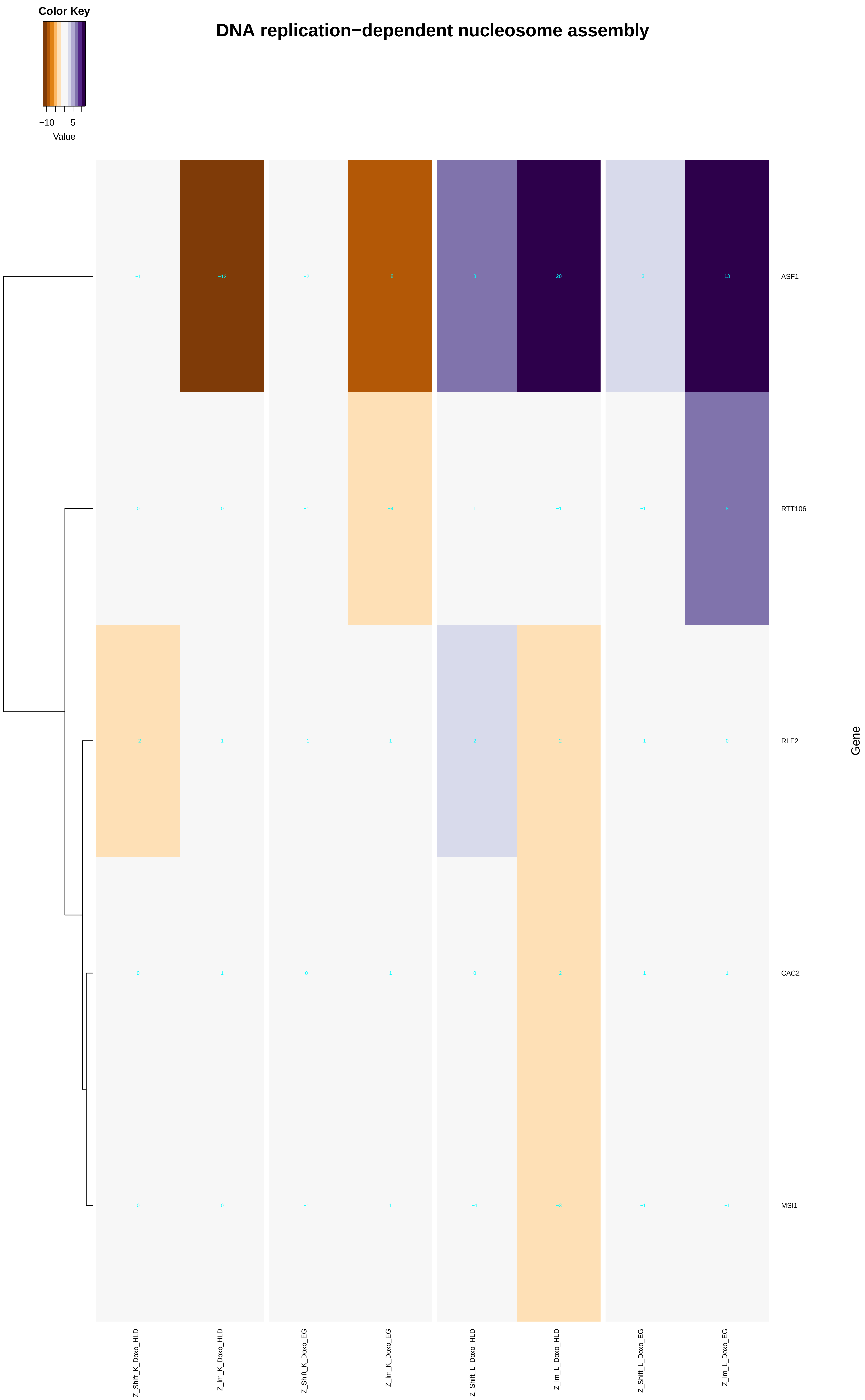

Color Key
−10
5
Value
DNA replication−dependent nucleosome assembly
−1
−12
−2
−8
8
20
3
13
ASF1
0
0
−1
−4
1
−1
−1
8
RTT106
−2
1
−1
1
2
−2
−1
0
RLF2
0
1
0
1
0
−2
−1
1
CAC2
0
0
−1
1
−1
−3
−1
−1
MSI1
Gene
Z_Shift_K_Doxo_HLD
Z_Im_K_Doxo_HLD
Z_Shift_K_Doxo_EG
Z_Im_K_Doxo_EG
Z_Shift_L_Doxo_HLD
Z_Im_L_Doxo_HLD
Z_Shift_L_Doxo_EG
Z_Im_L_Doxo_EG

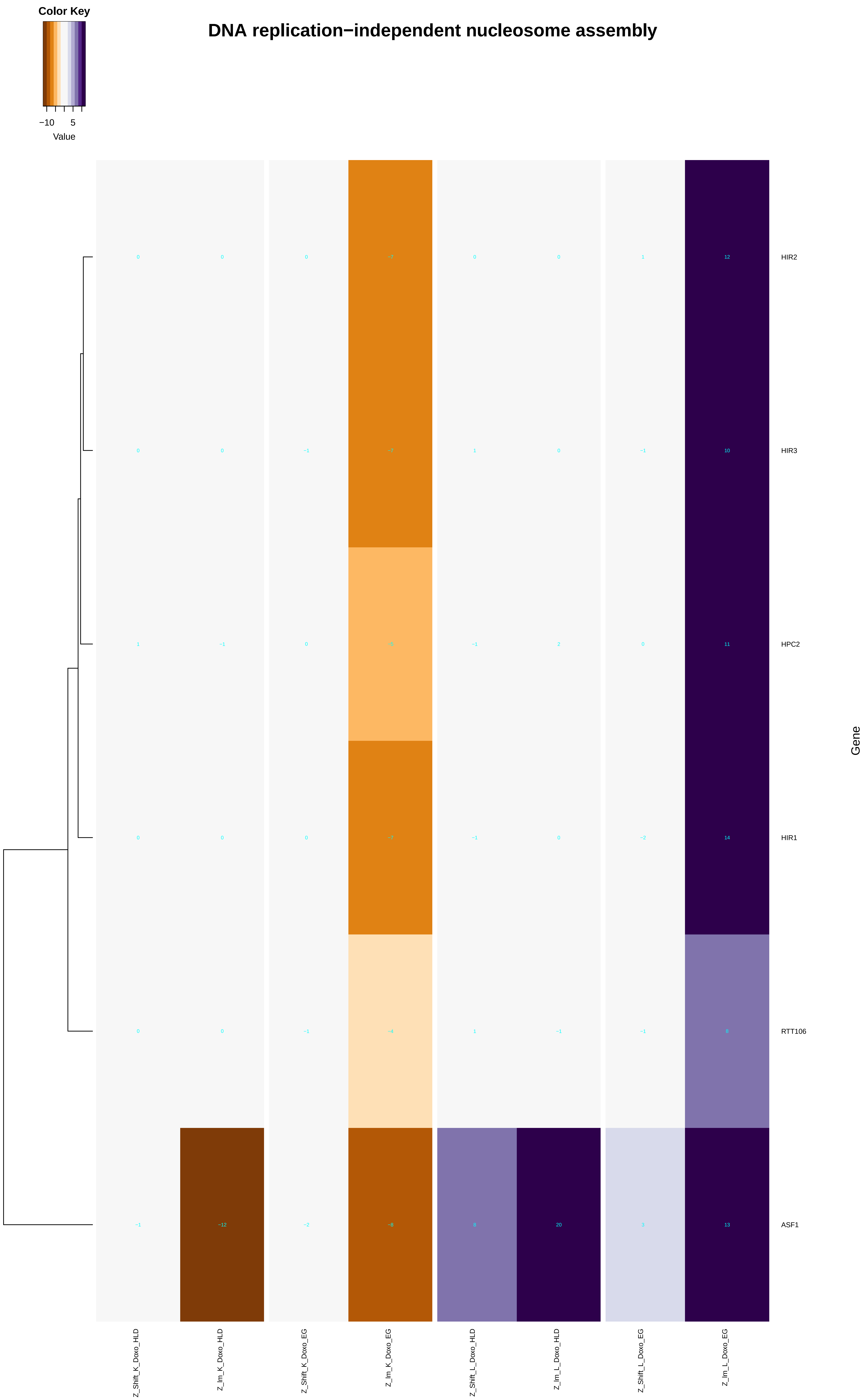
Color Key
−10
5
Value
DNA replication−independent nucleosome assembly
HIR2
HIR3
HPC2
HIR1
RTT106
ASF1
Gene
Z_Shift_K_Doxo_HLD
Z_Im_K_Doxo_HLD
Z_Shift_K_Doxo_EG
Z_Im_K_Doxo_EG
Z_Shift_L_Doxo_HLD
Z_Im_L_Doxo_HLD
Z_Shift_L_Doxo_EG
Z_Im_L_Doxo_EG

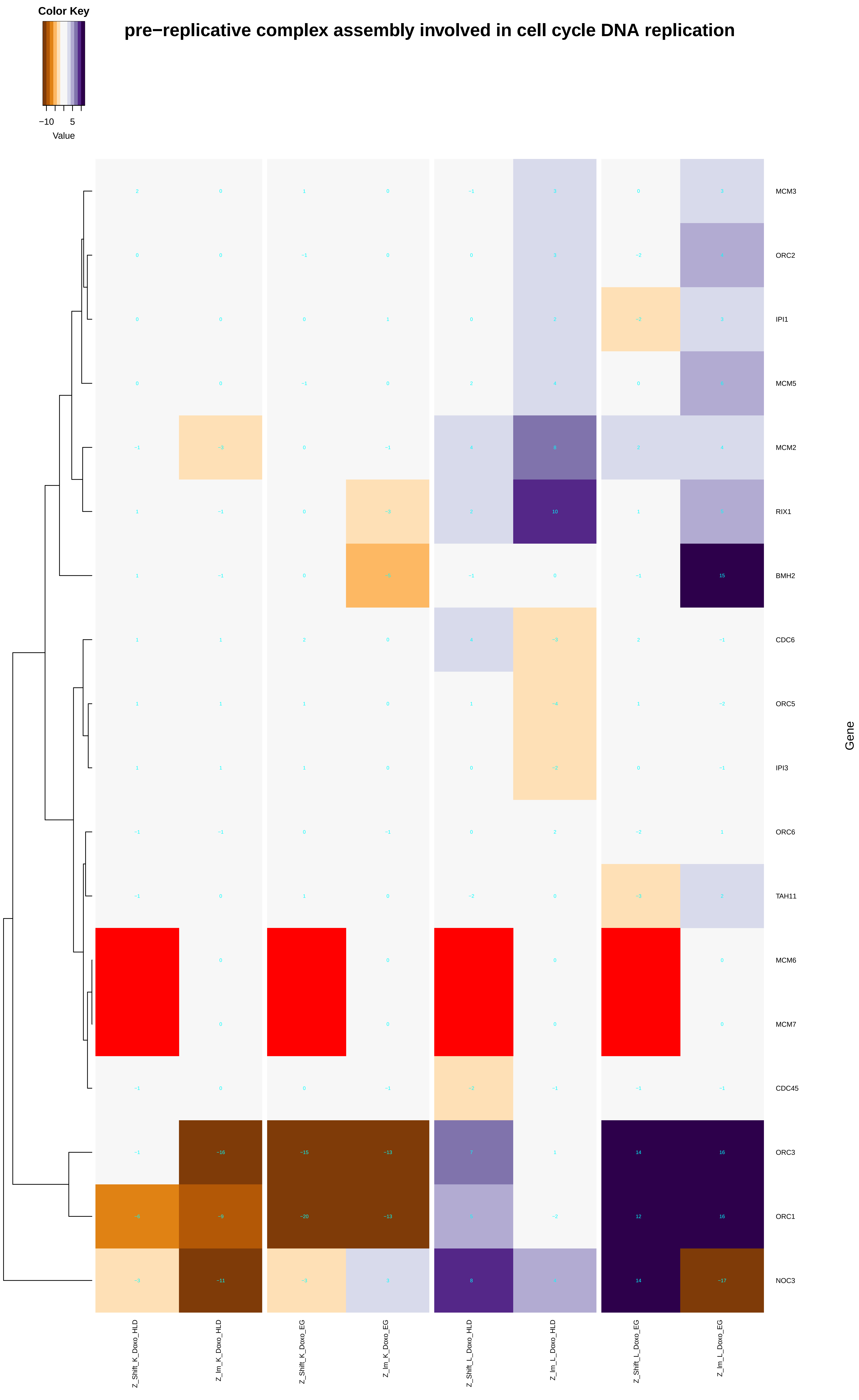

Color Key
−10
5
Value
pre−replicative complex assembly involved in cell cycle DNA replication
MCM3
ORC2
IPI1
MCM5
MCM2
RIX1
BMH2
CDC6
ORC5
IPI3
ORC6
TAH11
MCM6
MCM7
CDC45
ORC3
ORC1
NOC3
Gene
Z_Shift_K_Doxo_HLD
Z_lm_K_Doxo_HLD
Z_Shift_K_Doxo_EG
Z_lm_K_Doxo_EG
Z_Shift_L_Doxo_HLD
Z_lm_L_Doxo_HLD
Z_Shift_L_Doxo_EG
Z_lm_L_Doxo_EG

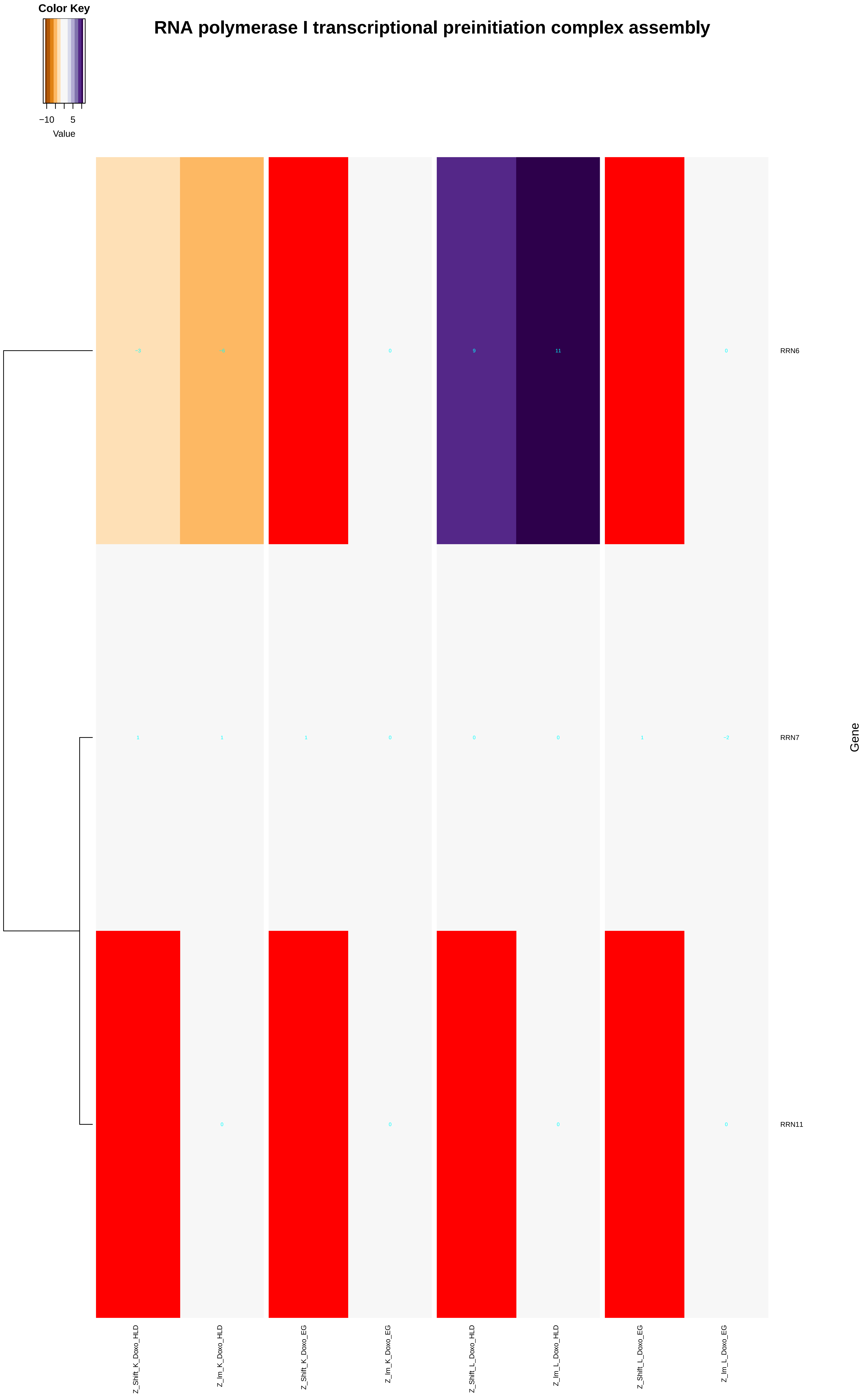

Color Key
−10
5
Value
RNA polymerase I transcriptional preinitiation complex assembly
−3
−6
0
9
11
0
1
1
1
0
0
0
1
−2
0
0
0
0
RRN6
RRN7
RRN11
Gene
Z_Shift_K_Doxo_HLD
Z_Im_K_Doxo_HLD
Z_Shift_K_Doxo_EG
Z_Im_K_Doxo_EG
Z_Shift_L_Doxo_HLD
Z_Im_L_Doxo_HLD
Z_Shift_L_Doxo_EG
Z_Im_L_Doxo_EG

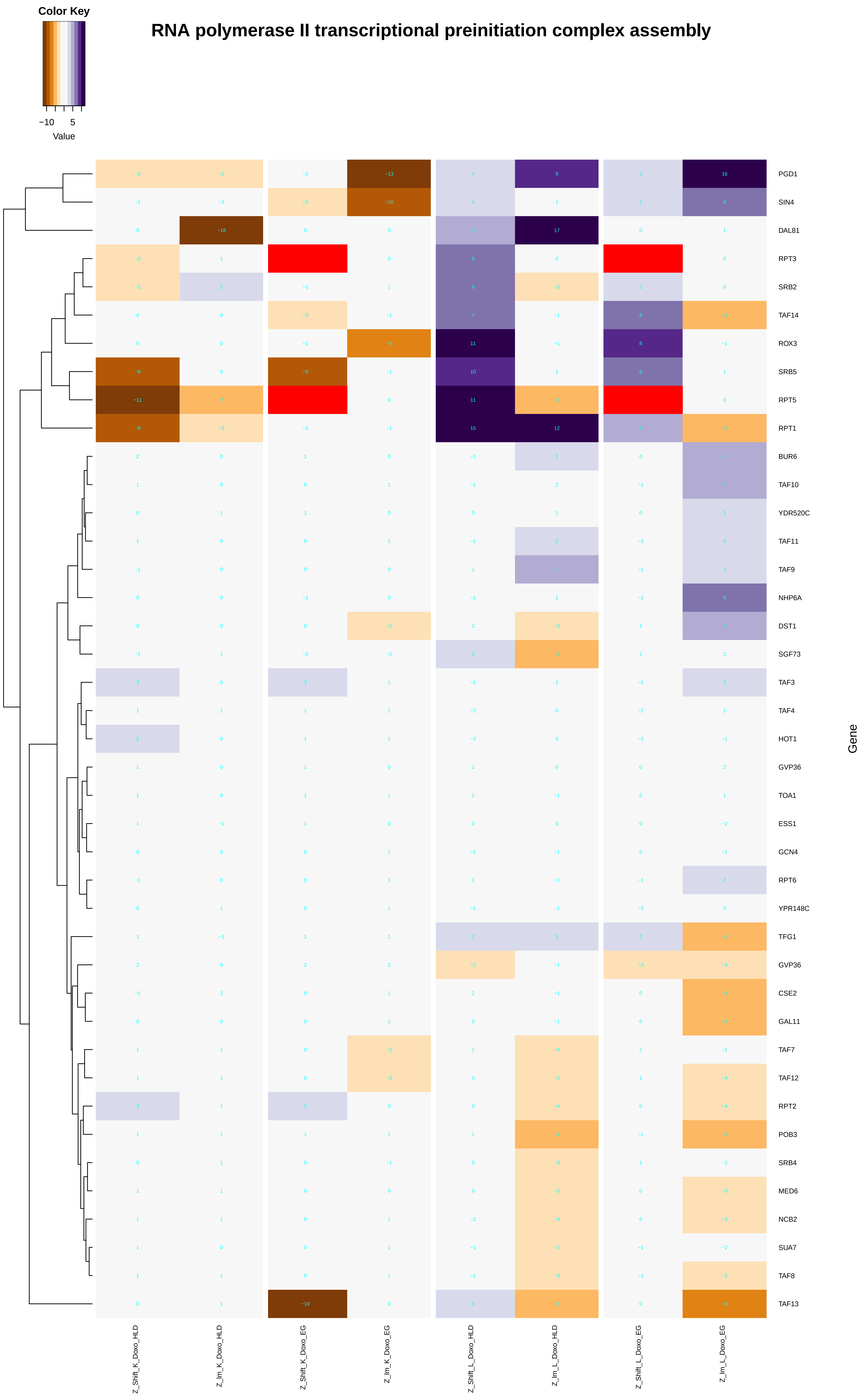
RNA polymerase II transcriptional preinitiation complex assembly
Color Key
−10
5
Value
Gene
PGD1
SIN4
DAL81
RPT3
SRB2
TAF14
ROX3
SRB5
RPT5
RPT1
BUR6
TAF10
YDR520C
TAF11
TAF9
NHP6A
DST1
SGF73
TAF3
TAF4
HOT1
GVP36
TOA1
ESS1
GCN4
RPT6
YPR148C
TFG1
GVP36
CSE2
GAL11
TAF7
TAF12
RPT2
POB3
SRB4
MED6
NCB2
SUA7
TAF8
TAF13
Z_Shift_K_Doxo_HLD
Z_Im_K_Doxo_HLD
Z_Shift_K_Doxo_EG
Z_Im_K_Doxo_EG
Z_Shift_L_Doxo_HLD
Z_Im_L_Doxo_HLD
Z_Shift_L_Doxo_EG
Z_Im_L_Doxo_EG

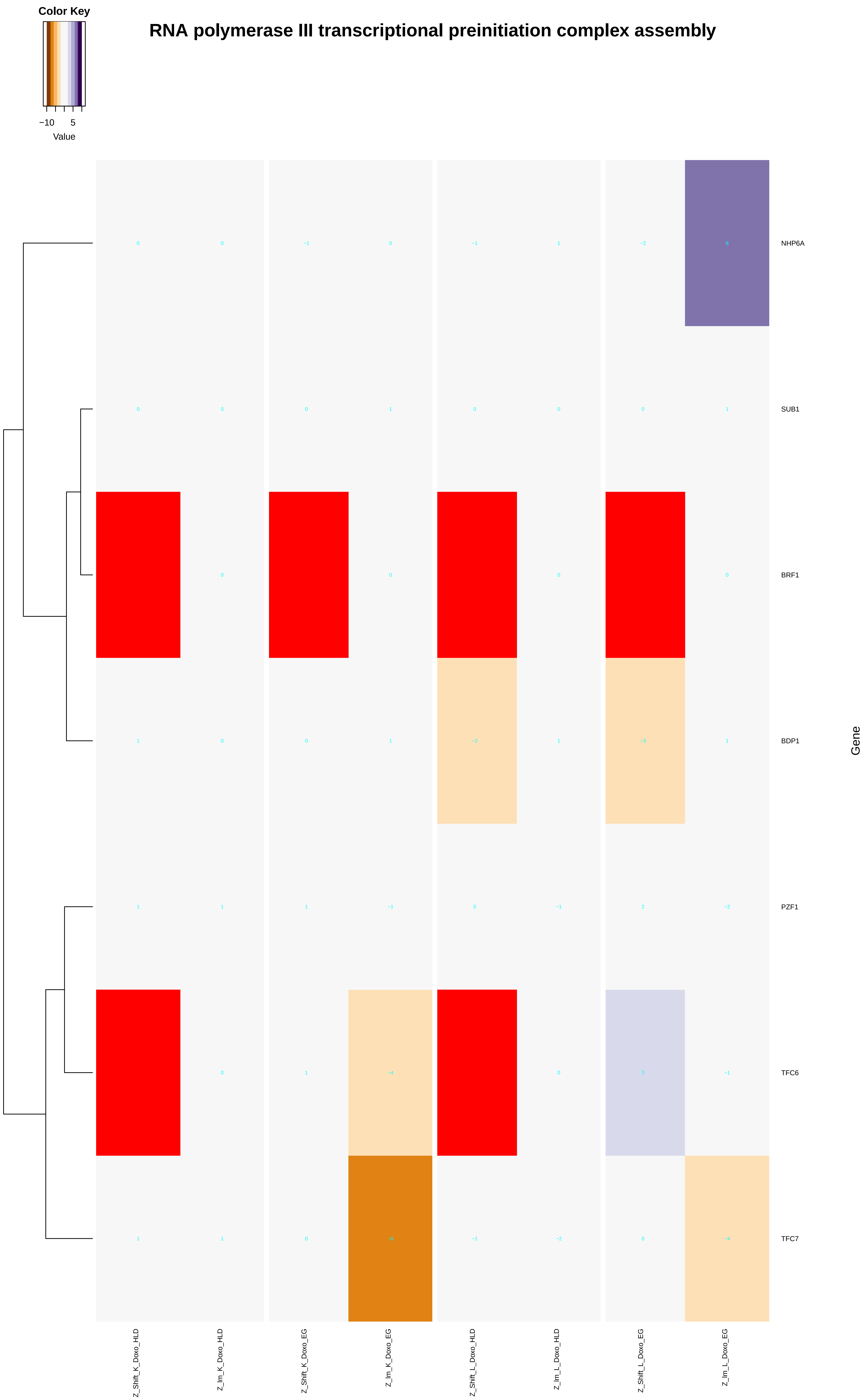
RNA polymerase III transcriptional preinitiation complex assembly
Color Key
−10
5
Value
NHP6A
SUB1
BRF1
BDP1
PZF1
TFC6
TFC7
Gene
Z_Shift_K_Doxo_HLD
Z_Im_K_Doxo_HLD
Z_Shift_K_Doxo_EG
Z_Im_K_Doxo_EG
Z_Shift_L_Doxo_HLD
Z_Im_L_Doxo_HLD
Z_Shift_L_Doxo_EG
Z_Im_L_Doxo_EG

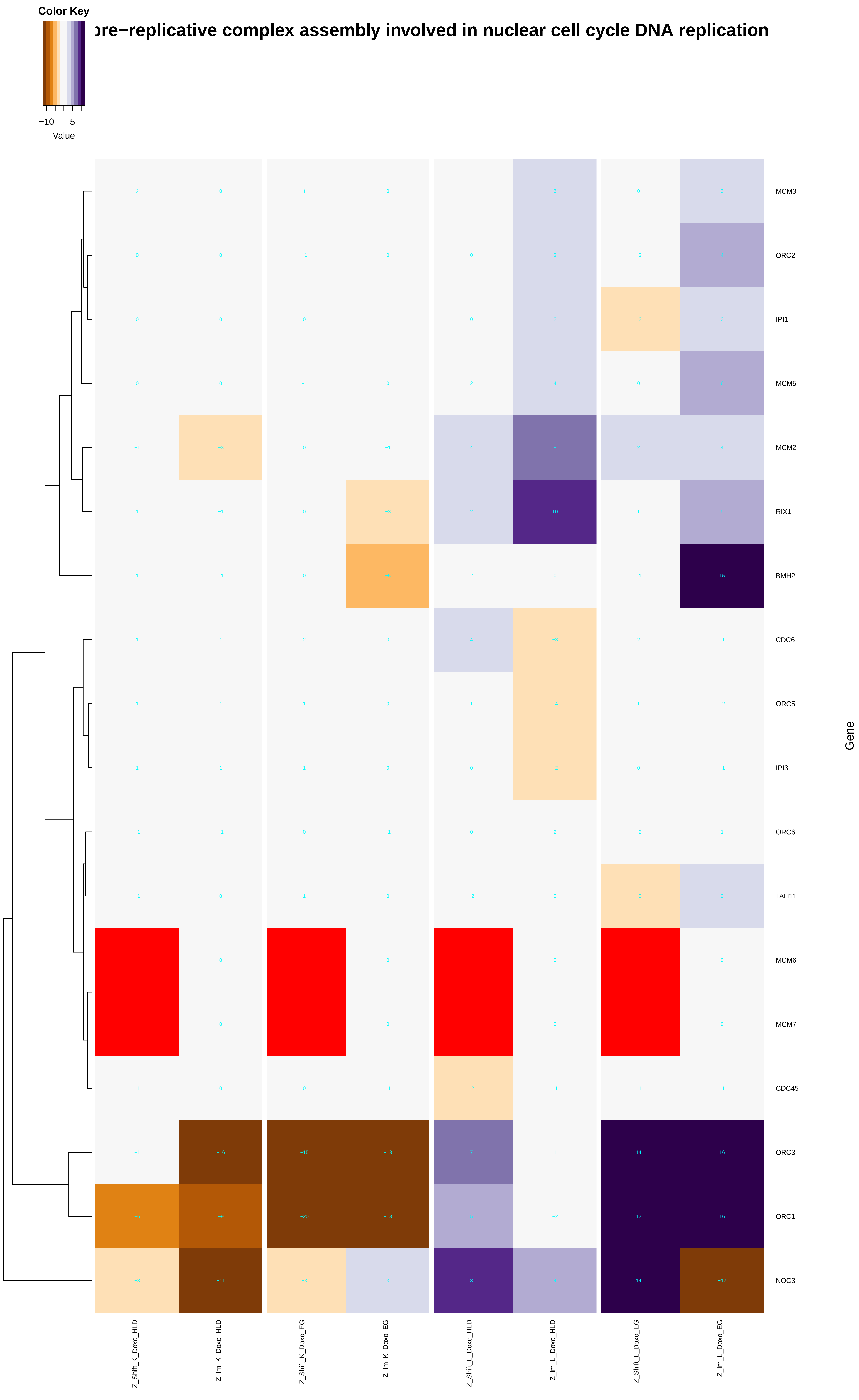

Color Key
−10
5
Value
ɔre−replicative complex assembly involved in nuclear cell cycle DNA replication
MCM3
ORC2
IPI1
MCM5
MCM2
RIX1
BMH2
CDC6
ORC5
IPI3
ORC6
TAH11
MCM6
MCM7
CDC45
ORC3
ORC1
NOC3
Gene
Z_Shift_K_Doxo_HLD
Z_Im_K_Doxo_HLD
Z_Shift_K_Doxo_EG
Z_Im_K_Doxo_EG
Z_Shift_L_Doxo_HLD
Z_Im_L_Doxo_HLD
Z_Shift_L_Doxo_EG
Z_Im_L_Doxo_EG

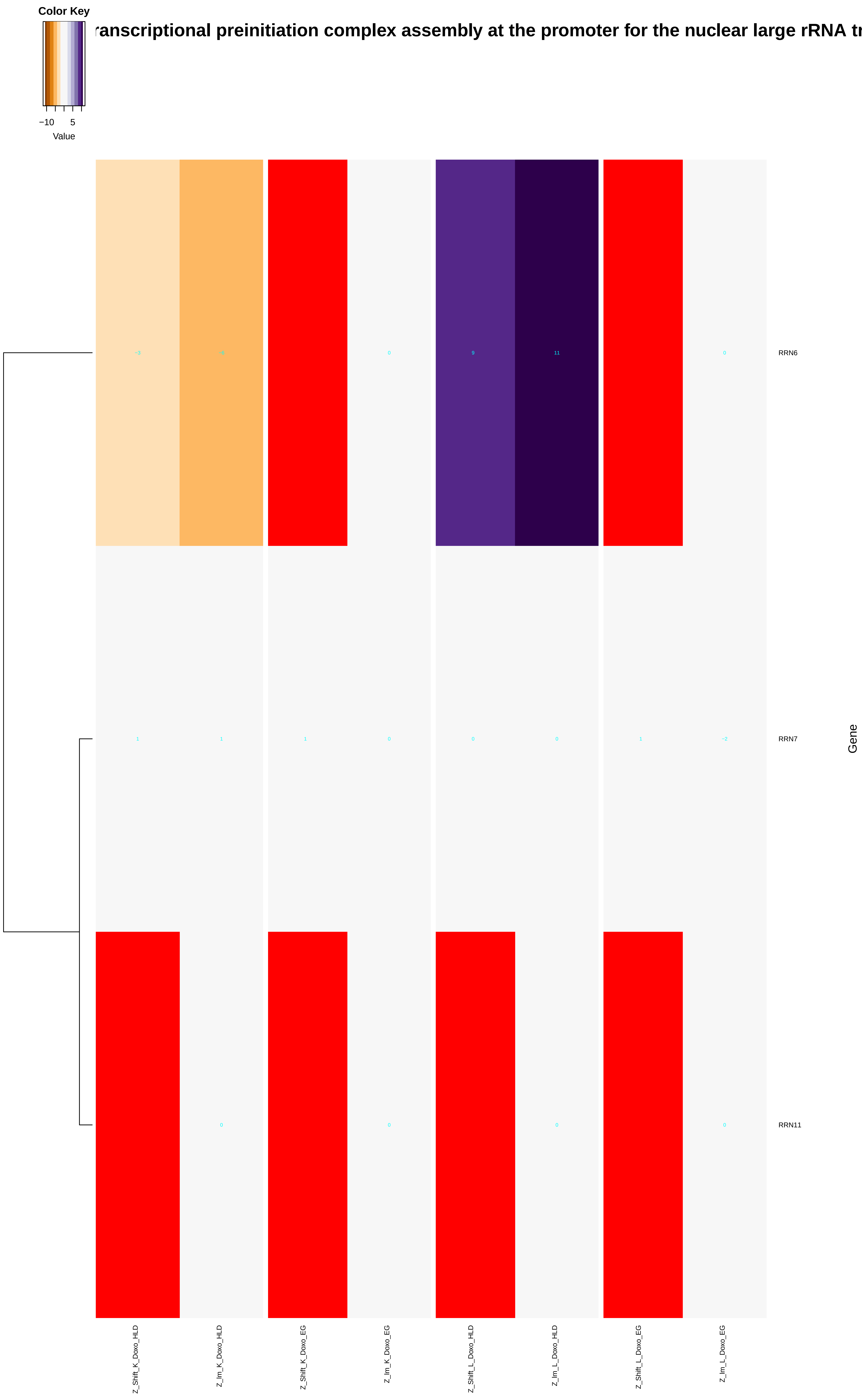

Color Key
−10
5
Value
ranscriptional preinitiation complex assembly at the promoter for the nuclear large rRNA tr
−3
−6
0
9
11
0
RRN6
1
1
1
0
0
0
1
−2
RRN7
0
0
0
0
RRN11
Gene
Z_Shift_K_Doxo_HLD
Z_Im_K_Doxo_HLD
Z_Shift_K_Doxo_EG
Z_Im_K_Doxo_EG
Z_Shift_L_Doxo_HLD
Z_Im_L_Doxo_HLD
Z_Shift_L_Doxo_EG
Z_Im_L_Doxo_EG

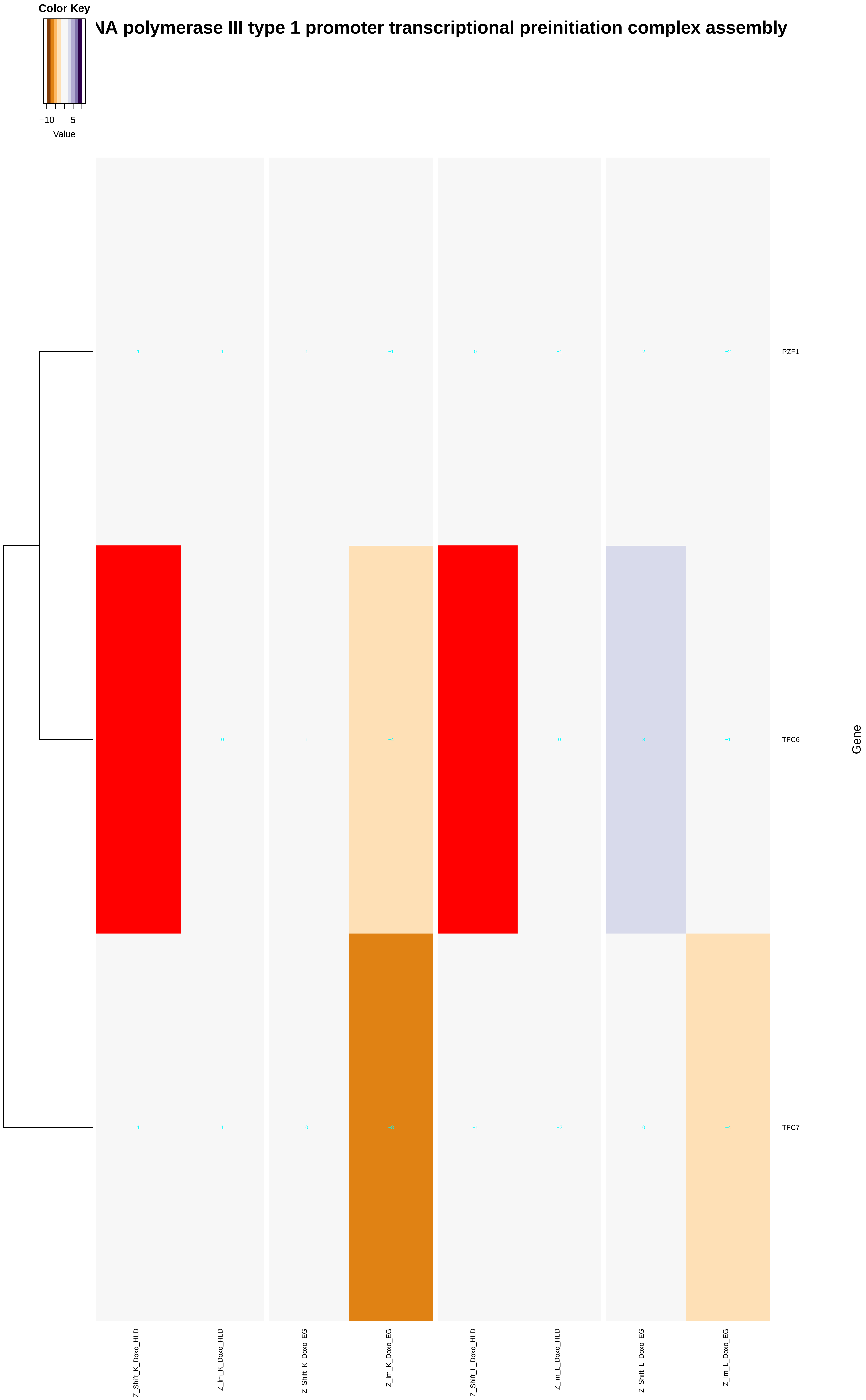

Color Key
NA polymerase III type 1 promoter transcriptional preinitiation complex assembly
−10
5
Value
PZF1
TFC6
TFC7
Gene
Z_Shift_K_Doxo_HLD
Z_Im_K_Doxo_HLD
Z_Shift_K_Doxo_EG
Z_Im_K_Doxo_EG
Z_Shift_L_Doxo_HLD
Z_Im_L_Doxo_HLD
Z_Shift_L_Doxo_EG
Z_Im_L_Doxo_EG
